# Letter by Kong et al. Regarding Article, “Is FAM19A5 an adipokine? Peripheral FAM19A5 in wild-type, FAM19A5 knockout, and LacZ knockin mice”

**DOI:** 10.1016/j.mocell.2025.100289

**Published:** 2025-10-22

**Authors:** Wei Kong

**Affiliations:** Department of Physiology and Pathophysiology, School of Basic Medical Sciences, Peking University; State Key Laboratory of Vascular Homeostasis and Remodeling, Peking University, Beijing 100191, China

To the Editor,

The recent study by Hoyun Kwak et al*.*, titled *“Is FAM19A5 an adipokine? Peripheral FAM19A5 in wild-type, FAM19A5 knockout, and LacZ knockin mice”* (Kwak et al., 2024), presents the possible distribution of FAM19A5 expression across different organs or tissues and reports a novel potential receptor that binds to FAM19A5. By leveraging Laz knockin technology to trace FAM19A5 in mice, the authors claim that FAM19A5 is not expressed in adipose tissue. However, upon closer examination, several significant issues and limitations need deep discussion, particularly concerning the technical methods used for the purification of FAM19A5 protein and the ELISA assay for measuring FAM19A5 levels.

The first confusing data is that the relative quantity of *Fam19a5*-isofrom 2 mRNA is truly expressed in white adipose tissue: 87.2 in males and 91.9 in females. In other organs, relative *Fam19a5*-isofrom 2 mRNA expression levels are 24.6 in males and 22.9 in females in the heart tissue, 5.2 in males and 4.2 in females in the intestine, and 60.1 in males’ testes as shown in their study. Undoubtedly, white adipose tissue exhibits significantly higher expression of FAM19A5 compared to these organs or tissues. However, it is unclear why the authors detected the X-gal positive staining in heart, small intestine, and testis, but not in white adipose tissue in Laz knock-in mice. These inconsistent results may have certain constraints under specific conditions, which raises doubts about the evidence to exclude the expression of FAM19A5 in adipose tissue.

Another concern is that in WT mice, the authors observed only negligible levels of FAM19A5 both in peripheral adipose tissue and plasma via ELISA. However, the ELISA used in Hoyun Kwak et al.’s work is not a canonical double-antibody sandwich method. Surprisingly, they used a 6xHis-TEV-LRRC4B (453-576) protein, but not an anti-FAM19A5 antibody to coat a 96-well plate, which is not suitable for ELISA. The secondary antibody the authors used to conjugate HRP is harvested from immunized with purified N-terminal His-tagged FAM19A5 protein, which is probably not a mature and secreted FAM19A5. Meanwhile, this paper does not reveal the recognition epitope sequence of anti-FAM19A5 antibody. In line with the standard operating procedure of ELISA (Engvall et al., 1971), both the capture antibody and the secondary antibody must be antibodies to specifically against the FAM19A5, but not other proteins (6xHis-TEV-LRRC4B in this paper) to potentially interact with FAM19A5.

In contrast to the findings reported in this article, substantial evidence supports the notion that FAM19A5 acts as an adipokine secreted by adipose tissue, as demonstrated by our previous work and other groups (Huang et al., 2020, Wang et al., 2018). In our previous work, we applied TaqMan real-time PCR to analyze FAM19A5 expression in human tissues by using a human cDNA library and verified its adipose expression by immunohistochemistry staining in human, rat, and mouse adipose tissue using commercially available antibody (R&D Systems). We also detected secreted FAM19A5 levels in the supernatant of cultured adipose tissue/cells with or without several stimulators, including inflammatory cytokines and insulin. Furthermore, we tested FAM19A5 levels in the plasma of WT/transgenic mice with or without HFD by ELISA and Cytometric Bead Assay, respectively (Wang et al., 2018). Additionally, we further reanalyzed signal nuclei RNA sequence (sn-RNA seq) of human adipose tissue (GSM5699630) (Whytock et al., 2022). Integration and clustering of whole adipose tissue sample yielded 7 clusters from 2,749 nuclei (Fig. 1A). Adipocytes were identified as *PERILIPIN-1* positive cells (an adipocyte marker) (Fig. 1B, C). Notably, *FAM19A5* is also expressed in the *Perilipin-1* positive clusters (Fig. 1D).

**Fig. 1:**
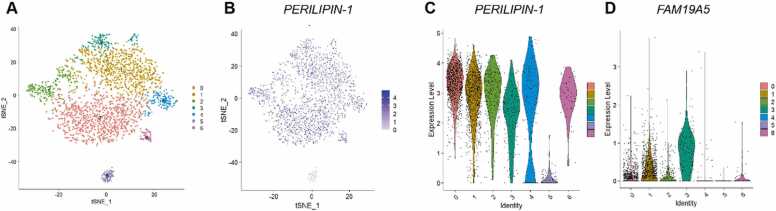
Single nuclei RNA-Seq of human subcutaneous adipose tissue (WAT). (A) UMAP showing 6 clusters from 2,749 nuclei. (B, C) *PERILIPIN-1* expression between different clusters. (D) Violin plot showing *FAM19A5* expression in *PERILIPIN-1–*expressed clusters.

Taken together, these published data demonstrate that, in addition to its higher expression in the central nervous system, FAM19A5 is also an adipokine that is expressed and secreted from adipose tissue.

Second, the authors claim that S1PR2 is not the receptor to bind FAM19A5. They mentioned in the method section that they used a His-tag at N-terminal of FAM19A5 for protein purification, antibody preparation, and subsequent receptor assay, but without the detailed information regarding the fusion site of His-tag at N-terminus. FAM19A5 is a chemokine [C-C motif]-like cytokine, which belongs to a family with sequence similarity 19 composed of 5 highly homologous genes (FAM19A1-5). As a cytokine, mature and secreted FAM19A5 does not contain an N-terminal signal peptide (N-terminal) (Tang et al., 2004, Wang et al., 2018). Thus, the fusion site is critical. If the His-tag fused at the N-terminus of signal peptide, the protein they obtained would be the signal peptide of FAM19A5, rather than the mature secretory form of the peptide. Since there is no detailed information in this study showing the amino acids sequence and bioactivity of purified FAM19A5, particularly, the paper did not include the detailed regarding how to incorporate the His-tag at N-terminus, it is possible that they did not find FAM19A5 binds to S1PR2 and activates its downstream Gi and β-arrestin signaling due to only administration of FAM19A5 signal peptide rather than mature full length FAM19A5. Therefore, the feasibility of purifying mature and secretory FAM19A5 with a His-tag at N-terminus remains seriously questionable. In our previous work, we have applied radio-ligand binding assay (a gold standard for determining the receptor-ligand interaction), co-immunoprecipitation, receptor internalization, Ca^2+^ mobilization, G12/13 RhoA activation, and VSMCs functional assay to cumulatively demonstrate that FAM19A5 is the ligand of S1PR2 in VSMCs. Then, we have found that the FAM19A5 elicited the S1PR2-G12/G13 signaling in VSMCs (Wang et al., 2018).

In summary, Kwak et al’s study indicates the probable distribution of FAM19A5 crossing various organs or tissues, but it probably raises some critical scientific concerns, including the inconsistent adipose FAM19A5 expression data, the potentially unreliable ELISA measurement, as well as the unclear information of N-terminal His-tagged FAM19A5 they purified, which insufficiently support the conclusion. Thus, this research may lead to several potential scopes for further discussion in the study of FAM19A5.

## Funding and Support

This research was supported by funding from the 10.13039/501100001809National Natural Science Foundation of China (NSFC, 31930056 and 81921001).

## Author Contributions

**Wei Kong:** Writing – review & editing, Writing – original draft, Resources, Project administration, Investigation, Conceptualization.

## Declaration of Competing Interests

The author declares that they have no known competing financial interests or personal relationships that could have appeared to influence the work reported in this paper.
